# Supplementary material for: Sex dimorphism in European sea bass (Dicentrarchus labrax L.): New insights into sex-related growth patterns during very early life stages
Source: PLoS One. 2021 Apr 22;16(4):e0239791. doi: 10.1371/journal.pone.0239791 (PMC8061996; doi:10.1371/journal.pone.0239791)
Supplement: S1 Fig — (PDF) [file pone.0239791.s001.pdf]

## Supplementary material 1. Image analysis to recover length, height, perimeter and area of the fish

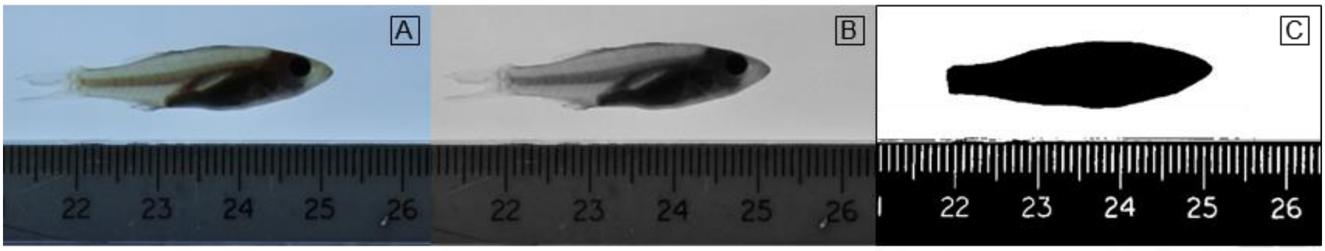


**Fig. S1.** Example of image analysis of a 110 dph (707 degree days above 10 °C) fish, following de Verdal et al. (2014): A) original image, B) 8-bit image, C) adjustment of the threshold to blacken only the fish and proceed to the analysis of pixel particles to recover length, height, perimeter and area. All the steps were performed through ImageJ software 1.51 (Rasband, 1997-2018).
