# Supplementary material for: Sex dimorphism in European sea bass (Dicentrarchus labrax L.): New insights into sex-related growth patterns during very early life stages
Source: PLoS One. 2021 Apr 22;16(4):e0239791. doi: 10.1371/journal.pone.0239791 (PMC8061996; doi:10.1371/journal.pone.0239791)
Supplement: S2 Fig — (PDF) [file pone.0239791.s002.pdf]

Supplementary material 2. Determination of the sex of the fish

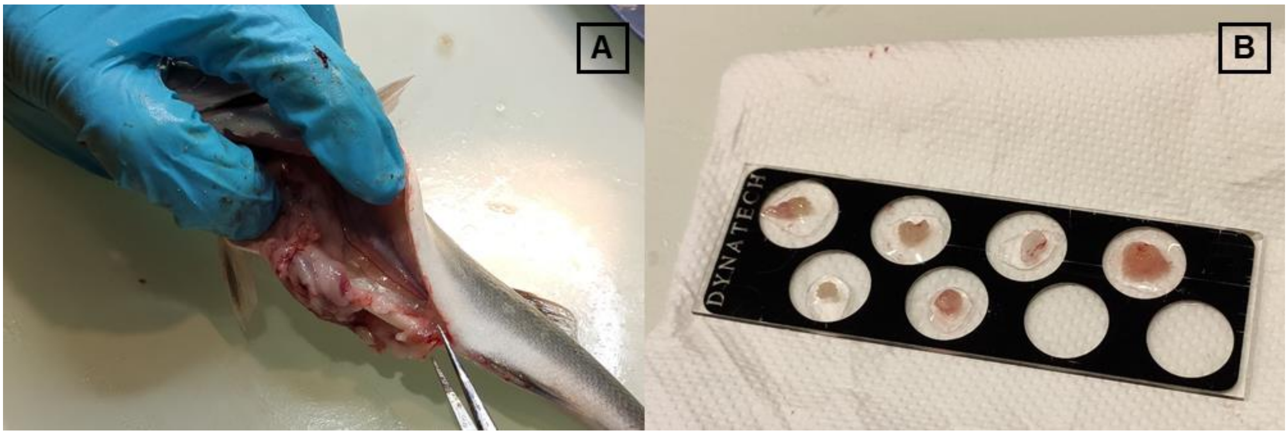

Fig. S2. Determination of the sex of 358 dph (3229 degree days above 10 °C) fish, either through macroscopically observation of the gonads (A) or gonadal squash (B) following Menu et al. (2005).
