# Supplementary material for: Sex dimorphism in European sea bass (Dicentrarchus labrax L.): New insights into sex-related growth patterns during very early life stages
Source: PLoS One. 2021 Apr 22;16(4):e0239791. doi: 10.1371/journal.pone.0239791 (PMC8061996; doi:10.1371/journal.pone.0239791)
Supplement: S4 Fig — (PDF) [file pone.0239791.s004.pdf]

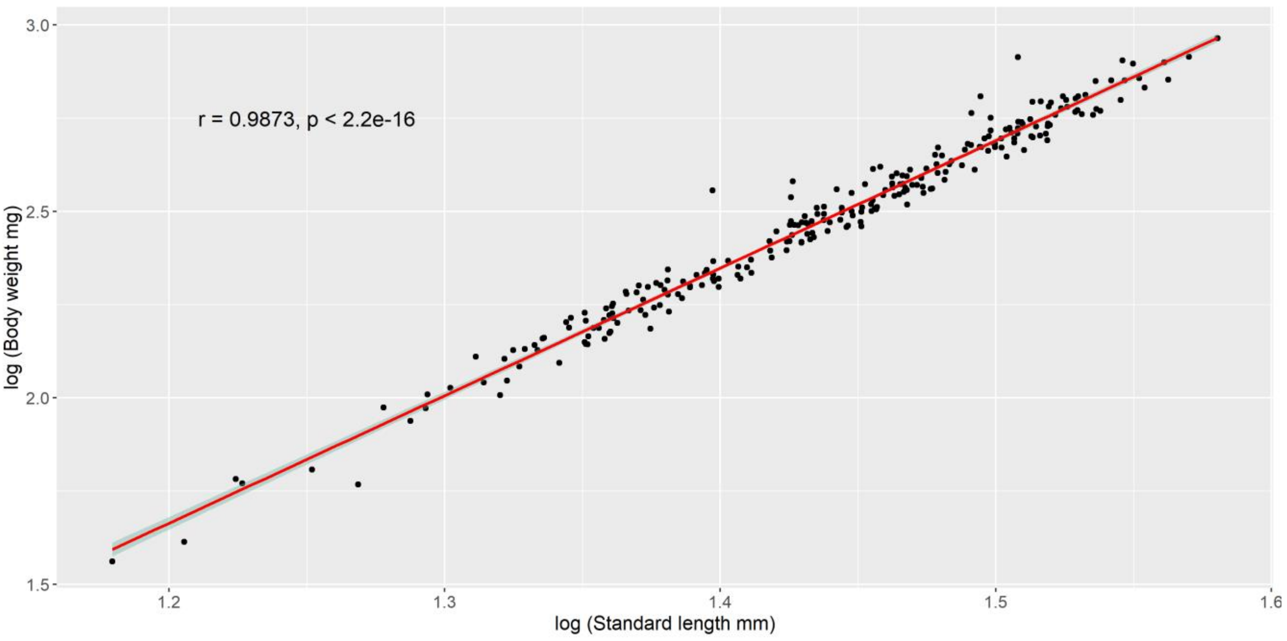

Fig. S4. Regression between logarithm of the measured standard length and logarithm of the measured body weight.
