# Supplementary material for: Sex dimorphism in European sea bass (Dicentrarchus labrax L.): New insights into sex-related growth patterns during very early life stages
Source: PLoS One. 2021 Apr 22;16(4):e0239791. doi: 10.1371/journal.pone.0239791 (PMC8061996; doi:10.1371/journal.pone.0239791)
Supplement: S5 Fig — (PDF) [file pone.0239791.s005.pdf]

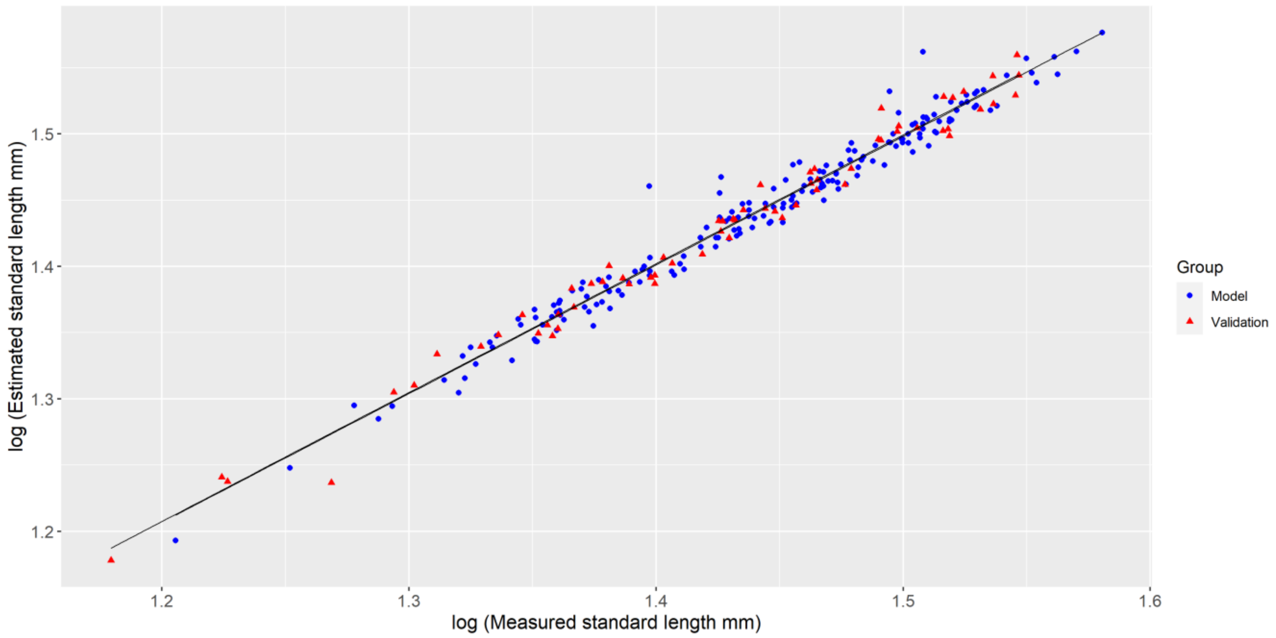

Fig. S5. Regression between logarithm of measured and estimated values of fish standard length using the model (2) for the "model set" (blue circles) and the "validation set" (red triangles).
