# Supplementary material for: Sex dimorphism in European sea bass (Dicentrarchus labrax L.): New insights into sex-related growth patterns during very early life stages
Source: PLoS One. 2021 Apr 22;16(4):e0239791. doi: 10.1371/journal.pone.0239791 (PMC8061996; doi:10.1371/journal.pone.0239791)
Supplement: S1 Table — (PDF) [file pone.0239791.s006.pdf]

Supplementary material 3. Prediction of body weight from digital picture measurements and prediction of standard length

Table S1

Pearson's coefficient of correlation (r) between traits (N = 250). Area, perimeter, height, length: obtained from image analysis; volume: calculated using the formula detailed in the manuscript; SL: measured standard length; BW: measured body weight. Asterisks indicate significant correlations (*p*-value < .0001 '****').

Trait	Area	Perimeter	Height	Length	Volume	SL	BW
Area		0.9859****	0.9901****	0.9842****	0.9913****	0.9807****	0.9898****
Perimeter			0.9780****	0.9894****	0.9628****	0.9822****	0.9604****
Height				0.9738****	0.9780****	0.9725****	0.9744****
Length					0.9552****	0.9920****	0.9558****
Volume						0.9533****	0.9963****
