# Supplementary material for: Sex dimorphism in European sea bass (Dicentrarchus labrax L.): New insights into sex-related growth patterns during very early life stages
Source: PLoS One. 2021 Apr 22;16(4):e0239791. doi: 10.1371/journal.pone.0239791 (PMC8061996; doi:10.1371/journal.pone.0239791)
Supplement: S2 Table — (PDF) [file pone.0239791.s007.pdf]

Table S2

Coefficient of determination (R^2) and Akaike information criterion (AIC) for models of multiple regression to estimate body weight.

N. of traits	Model	R^2	AIC
3	Area + Volume + Perim	0.9942	-1053.5
2	Area + Volume	0.9939	-1046.0
4	Area + Perim + Height + Length	0.9938	-1042.0
3	Area + Length + Height	0.9936	-1037.1
2	Perim + Volume	0.9936	-1037.3
1	Volume	0.9935	-1036.8
3	Area + Length + Perim	0.9922	-1001.2
2	Area + Length	0.9918	-991.6
3	Area + Perim + Height	0.9894	-942.9
2	Area + Perim	0.9883	-926.6
2	Area + Height	0.9822	-848.2
1	Area	0.9802	-830.1
2	Perim + Height	0.9524	-666.4
3	Length + Height + Perim	0.9522	-664.7
1	Height	0.9522	-666.9
2	Length + Height	0.9521	-665.3
2	Perim + Length	0.9227	-576.8
1	Perim	0.9226	-577.7
1	Length	0.9108	-551.3
