# Supplementary material for: Sex dimorphism in European sea bass (Dicentrarchus labrax L.): New insights into sex-related growth patterns during very early life stages
Source: PLoS One. 2021 Apr 22;16(4):e0239791. doi: 10.1371/journal.pone.0239791 (PMC8061996; doi:10.1371/journal.pone.0239791)
Supplement: Attachment — Submitted filename: PONE-D-20-28397_review.pdf [file pone.0239791.s008.pdf]

Review of PONE-D-20-28397 for Plos One

"Sex dimorphism in European sea bass (Dicentrarchus labrax L.): new insights into sex related growth patterns during very early life stages", by Faggion et al.

This manuscript examines the differences in growth trajectories between the two sexes of European sea bass. Given the well-known association between growth rate during the early life stages and sex ratio in this species (e.g. references cited herein), the authors used the latest advances in fish tagging methodology to follow individual growth trajectories from ca 23 to ca 177 mm SL. At the end of the study, fish were categorized according to their sex (following gonadal examination) and sex-specific growth rates were estimated. Sampling for body-size measurements was done regularly during the studied period (15 samplings in total, 83-335 dph). For the longest time interval (117-335 dph) reported growth data concerned 87 female and 45 male individuals, while for the earlier period (83-110 dph) data for 11-45 females and 7-26 males were recorded. Despite the high interest in the scientific question addressed, as well as the novelty of the used methodology for the monitoring of individual growth, data analysis methodology needs substantial improvement.

Major comments

1. Daily growth rates were estimated only for weight (and not for SL) by using a formula, which in not very common in the literature (instead e.g. of the formula based on the exponential growth model, (e.g. lnW2-lnW1/Dt)). As weight was directly measured on individual fish only after the 6$^{th}$ sampling (117 dph), DGR estimations for the earlier period (83-110 dph) were based on back-calculated weight estimations (based on estimated morphometric relationships). A DGR estimation on the primarily measured SL would better support the results of the ms on the higher growth rate of females at 96-103 dph period.
2. In many sampling points, body size was significantly larger in females than in males (Table 1). Despite this result, sex-related differences in DGR were significant only in one time period (96-103 dph, Table 2). The lack of agreement in these two approaches weakens the value of the findings. Especially because significant DGR differences were observed for a period where sample sizes were relatively small and not the same between the start and end points (96 and 103 dph respectively, Table 1, 2).
3. Given the availability of size-age data for a series of 15 sampling points, the estimation of growth curves on the basis of Ricker growth model (size=a*exp(r*age)) could solve the issue of comment No2, by involving a better statistical approach, with more accurate growth rate estimates. As during the studied period, different growth stanzas may exist, authors could use a) a piecewise linear regression to identify the inflexion points in the growth curves, and b) ANCOVA tests to compare the specific growth rate and “a” parameter between the two sexes (for each growth period separately).
   Such an approach is given in the figure below for the first growth period (based on the mean data provided in Table 1). The period of significant DGR differences is indicated by the circle. Arrow indicates a point of comparatively large residual value, which might partially explain the observed differences in the DGR for the period of 96-103 dph.

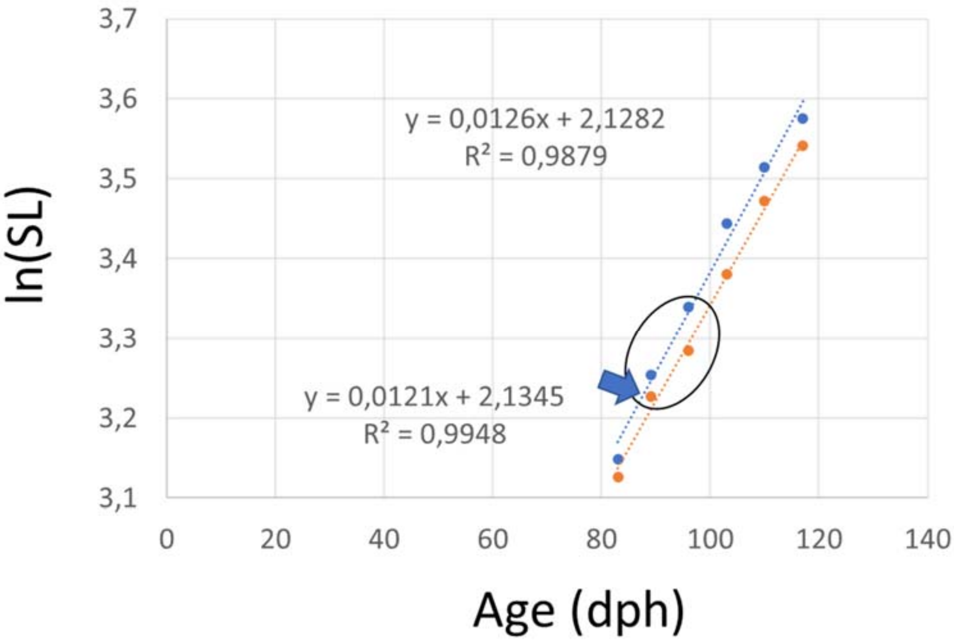


4. Growth models should be estimated for both length and weight. Also, I would suggest the authors to test sex-related differences in fish condition factor (e.g. Fulton's K).
5. Table S2. Fish volume was estimated as a function of length and depth (height, line 118). Therefore, its simultaneous use with length and height as independent variables in the multiple regression models (with W as dependent variable) statistically is not recommended.
6. Fish size and not age should be primarily used as indicative of ontogenetic progress, as well as for the comparison of the min fish size where SDD is reported by the different studies.
