# Supplementary material for: Sex dimorphism in European sea bass (Dicentrarchus labrax L.): New insights into sex-related growth patterns during very early life stages
Source: PLoS One. 2021 Apr 22;16(4):e0239791. doi: 10.1371/journal.pone.0239791 (PMC8061996; doi:10.1371/journal.pone.0239791)
Supplement: Attachment — Submitted filename: Response to Reviewers.docx [file pone.0239791.s009.docx]

In this rebuttal letter we answer to each point raised by the academic editor and reviewers.

**1. Please ensure that your manuscript meets PLOS ONE's style requirements, including those for file naming. The PLOS ONE style templates can be found at**

<https://journals.plos.org/plosone/s/file?id=wjVg/PLOSOne_formatting_sample_main_body.pdf>

<https://journals.plos.org/plosone/s/file?id=ba62/PLOSOne_formatting_sample_title_authors_affiliations.pdf>

We checked the manuscript to ensure that the journal’s style requirements were fulfilled. We formatted the headings, the tables, and the equations and we changed the affiliations and the references to tables, figures and supplementary materials to align them with PLOS ONE standards.

**2. In your Methods section, please include a comment about the state of the animals following this research. Were they euthanized or housed for use in further research?**

**If any animals were sacrificed by the authors, please include the method of euthanasia and describe any efforts that were undertaken to reduce animal suffering.**

We added a sentence describing the minimizing of animal suffering after each tagging surgery and when the animals were euthanized at the end of the experiment.

**3. Please include a copy of Table 3 which you refer to in your text on page 8.**

The table we refer to is indeed number 2 and not number 3; we corrected the reference.

**General comment**

While revising the data to answer the reviewers’ comments, we identified 4 fish with clear inconsistent BL and BW measurements at 103 and 110 dph. These fish did not have out of range measurement within a given date, and this is why we did not identify them previously, but their measurements between dates were not compatible. We removed these data from the analysis, resulting in slightly different means and SD for BW, SL and DGC at 103 and 110 dph.

**Reviewer #1: Major comments**

**1. Daily growth rates were estimated only for weight (and not for SL) by using a formula, which in not very common in the literature (instead e.g. of the formula based on the exponential growth model, (e.g. lnW2-lnW1/Dt)). As weight was directly measured on individual fish only after the 6th sampling (117 dph), DGR estimations for the earlier period (83-110 dph) were based on back-calculated weight estimations (based on estimated morphometric relationships). A DGR estimation on the primarily measured SL would better support the results of the ms on the higher growth rate of females at 96-103 dph period.**

DGC was preferred to SGR because it has been demonstrated to be more stable than SGR over the life of the fish and to linearly increase with temperature (Bureau et al, 2000; https://www.aquatech.com.ve/pdf/bureau.pdf). This makes possible the comparison between data from different periods of fish life, although even with DGC, different growth stanzas may exist (Dumas et al., 2007. *Aquaculture* 267, 139‑146).

DGC is not appropriate for SL. If fish do not change shape (Fulton’s K), the stability of DGC implies that growth in length (which is proportional to BW^1/3^) is linear according to temperature. Raising BL to ^(1/3) would create links between the residuals of the regression and age.

**2. In many sampling points, body size was significantly larger in females than in males (Table 1). Despite this result, sex-related differences in DGR were significant only in one time period (96-103 dph, Table 2). The lack of agreement in these two approaches weakens the value of the findings. Especially because significant DGR differences were observed for a period where sample sizes were relatively small and not the same between the start and end points (96 and 103 dph respectively, Table 1, 2).**

Since DGC estimation implies the starting and the final weight, for each age interval we considered the animals that had both initial and starting weight available. In the table, we reported the real number of animals for which the DGC estimation was possible. Since DGC is a growth rate, similar growth rates would result in the persistence of differences between males and females if they exist at the beginning of the period. Thus, we do not see this as a lack of agreement between the two methods.

**3. Given the availability of size-age data for a series of 15 sampling points, the estimation of growth curves on the basis of Ricker growth model (size=a*exp(r*age)) could solve the issue of comment No2, by involving a better statistical approach, with more accurate growth rate estimates. As during the studied period, different growth stanzas may exist, authors could use a) a piecewise linear regression to identify the inflexion points in the growth curves, and b) ANCOVA tests to compare the specific growth rate and “a” parameter between the two sexes (for each growth period separately). Such an approach is given in the figure below for the first growth period (based on the mean data provided in Table 1). The period of significant DGR differences is indicated by the circle. Arrow indicates a point of comparatively large residual value, which might partially explain the observed differences in the DGR for the period of 96-103 dph.**

We built the growth curves based on Ricker growth model for SL and BW, but we observed that this model seems to be not appropriate, as residuals were not even along the timeline. We instead built regressions of BW^1/3^ and untransformed SL on degree days above 10 °C, which is also coherent with the DGC approach. Doing this will yield similar results to the Ricker growth curves, but with a better fit.

**4. Growth models should be estimated for both length and weight. Also, I would suggest the authors to test sex-related differences in fish condition factor (e.g. Fulton’s K).**

Thanks for the comment. We estimated Fulton’s K condition factor and we performed statistical analyses to test sex-related differences. It is an added value to the paper, since it is the first time that Fulton’s K has been estimated in such early life stages of European sea bass (line 164-166).

**5. Table S2. Fish volume was estimated as a function of length and depth (height, line 118). Therefore, its simultaneous use with length and height as independent variables in the multiple regression models (with W as dependent variable) statistically is not recommended.**

Agree. We now use a model where height, length and volume were not simultaneously used, and we rearranged everything according to this change (line 192).

**6. Fish size and not age should be primarily used as indicative of ontogenetic progress, as well as for the comparison of the min fish size where SDD is reported by the different studies.**

Agree. We have always indicated both size and age (the first indication is the size, followed by the age), because we prefer to maintain the link with the tables (where the age is always indicated); in some cases, we also indicated the degree days.

**Reviewer #2: General comments**

**The work entitled “Sex dimorphism in European sea bass (*Dicentrarchus labrax* L.): new insights into sex-related growth patterns during very early life stages” studied sexual size dimorphism in European sea bass. There are three main concerns about the MS:**

**(1) The main weakness of the MS is lacking of histological observation of sex differentiation progress. Sex differentiation in fish is related to age (days post-fertilization), rearing conditions (e.g. temperature, nutrition), growth rate, and body weight/length. Without gonadal histology will make the results to be uncertain.**

The sexing has been performed at the end of the experiment (358 dph or 3229 degree days above 10 °C), when the identification through direct macroscopic observation of the gonads is possible and effective.

The study was not focused exactly on sex differentiation progress, but on the growth patterns of a batch of fish that were individually followed from a very early stage and, only at the end, recognized as males or females. Anyway, it is a very good point to discuss in a future perspective section and to implement in a new experiment.

**(2) There are usually three aspects of sex differentiation, cellular, morphological, and molecular sex differentiation. Molecular sex differentiation is much earlier than cellular and morphological sex differentiation, and is the cause of the other two. However, it is usually difficult to detect molecular sex differentiation until a late stage, because of the sampling problem of undifferentiated or differentiating gonad. Therefore, without the detect of molecular sex differentiation or morphological sex differentiation, the main conclusion “early growth may be a cause rather than a consequence of sex determination in sea bass” is unreliable.**

We proposed this as a hypothesis or a suggestion; the sentence was then modulated according to the reviewer’s comment (line 29).

**(3) Even though authors make progress in the SSD research using microchip tagging, the tagging process is also the main affecting factor of the results as authors discussed. Eliminating small sized fish during sorting for microchip tagging artificially altered the sex ratio of the experimental population. The sorting process may artificially advance and speed up the SSD because fast-growing females were dominating the population. In addition, as observed in the work, eliminating the most slow-growing male repressed the SSD. It may be the cause why the SSD was disappear in the late stage which is in contrast with previous reports.**

That is a possibility, and we have tried to discuss that. For the sake of clarity, we would like to underline that fish were not sorted before microchip tagging, fish were randomly chosen from a common garden tank and tagged (please see Faggion et al. 2020, doi: 10.1016/j.aquaculture.2020.734945). What we were discussing in the current paper was the fact that larvae were subjected to a “strong” manipulation (i.e. the microchip tagging), which might have acted itself as a sorting event, favoring stronger fish (that are likely to develop as females). Anyway, we have data from an “untagged” group, that was the control group of the previous work (see Faggion et al. 2020). In this previous paper, we have already reported that microchip tagging did not affect survival rates nor growth rates of the tagged group in comparison with the untagged group.

Looking at the sex ratio of the two groups (sex was recorded in all the individuals involved at the end of the experiment), this is the situation in the 2 groups:

- 65.6 % females : 34.4 % males in the tagged group, with a proportion of 1.91 females per male;
- 54.9 % females : 45.1 % males in the control “untagged” group, with a proportion of 1.22 females per male.

We did not find any significant differences in the proportion of males and females between groups (χ^2^ = 1.96, p-value = 0.16).

We rewrote this paragraph in the Discussion section (lines 347-353).

**Please also find some specific comments as follows.**

**Specific comments**

**1. Line 28: The term “sex determination” is misused here and thereafter in the following context.**

In European sea bass, sex determination does not happen at hatching but at some time after hatching, and it is both environmentally and genetically determined. Whether final sex depends on events that determine it before differentiation or to events happening during the initial phases of differentiation is not clear (see Vandeputte et al, 2020, https://onlinelibrary.wiley.com/doi/full/10.1002/ece3.6972). In this paper, we collected individual growth data in a batch of fish, and we used the final sex status to reconstruct the growth patterns of individuals that finally differentiated as males or females. When sex was recorded, it was already determined (as individuals were sexed at a stage of advanced juveniles), so we were not able to relate individuals to the sex differentiation process but only to the final sex status, hence the use of the term “sex determination”.

**2. It should be SSD, not SDD.**

True, it was a typo, we correct all the mistakes.

**3. Are there replicates for the experiments? It had not mentioned in the MS.**

The replicates are the individuals. In this work we were not testing different treatments, we have just performed individual observations of fish and all the fish were reared in the same environment, under the same conditions.

**4. Discussion section. The citation about an unpublished work again and again is not appropriate.**

We provided the correct citation since the work has now been published (see citation #26).
