# Supplementary material for: Sex dimorphism in European sea bass (Dicentrarchus labrax L.): New insights into sex-related growth patterns during very early life stages
Source: PLoS One. 2021 Apr 22;16(4):e0239791. doi: 10.1371/journal.pone.0239791 (PMC8061996; doi:10.1371/journal.pone.0239791)
Supplement: Attachment — Submitted filename: Response to Reviewers.docx [file pone.0239791.s010.docx]

**Review Comments to the Author**

**Reviewer #1: The authors have adequately responded to my comments. Given the existence of 5-6 previous papers on the topic (for the given species, the most of which have been already cited and discussed by the ms), I would recommend to the authors to include a line in the abstract stating that their results verify the results of previous studies.**

We have now included a sentence in the abstract to indicate that our results verify the results of previous studies (line 28).

**Reviewer #2: The authors had answered all my three inquiries but had revised nothing in the new MS version.**

The first comment of the reviewer in the previous round was the following: “**The main weakness of the MS is lacking of histological observation of sex differentiation progress. Sex differentiation in fish is related to age (days post-fertilization), rearing conditions (e.g. temperature, nutrition), growth rate, and body weight/length. Without gonadal histology will make the results to be uncertain”.**

For this, we precised that sexing was performed at the end of the experiment, so that our results in terms of sex of the fish (as at that stage sexing is not ambiguous) are not uncertain. We did not feel we had to modify the text in this case. In any case, the main interest of the study is to follow fish growth individually, knowing their final sexual fate. It would not be feasible to have (lethal) gonadal histology and keep this individual tracking of growth performance.

The second comment of the referee was the following: **“There are usually three aspects of sex differentiation, cellular, morphological, and molecular sex differentiation. Molecular sex differentiation is much earlier than cellular and morphological sex differentiation, and is the cause of the other two. However, it is usually difficult to detect molecular sex differentiation until a late stage, because of the sampling problem of undifferentiated or differentiating gonad. Therefore, without the detect of molecular sex differentiation or morphological sex differentiation, the main conclusion “early growth may be a cause rather than a consequence of sex determination in sea bass” is unreliable.”**

The full sentence was “This leads to the hypothesis that early growth may be a cause rather than a consequence of sex determination”. This is not a conclusion but clearly stated as an hypothesis. Even though, in the revised manuscript, we had replaced “may” by “might” to make the hypothetical nature of the statement even clearer. Even though this hypothesis is not proven, we feel important to formulate it as it may be the basis of future research.

The third comment of the referee was “**Even though authors make progress in the SSD research using microchip tagging, the tagging process is also the main affecting factor of the results as authors discussed. Eliminating small sized fish during sorting for microchip tagging artificially altered the sex ratio of the experimental population. The sorting process may artificially advance and speed up the SSD because fast-growing females were dominating the population. In addition, as observed in the work, eliminating the most slow-growing male repressed the SSD. It may be the cause why the SSD was disappear in the late stage which is in contrast with previous reports.”**

For this point, we added a whole paragraph in the discussion, lines 353-360: “In a previous paper [17], it was reported that microchip tagging did not affect survival rates nor growth rates of the tagged group in comparison with the untagged group. Analyzing the sex ratio of the tagged group (i.e. the fish of the current experiment) and the untagged group (i.e. the control group in [17]), we observed 65.6 % of females and 34.4 % of males in the first case, with a proportion of 1.91 females per male; 54.9 % of females and 45.1 % of males in the second case, with a proportion of 1.22 females per male. The proportion of males and females was not statistically different between groups (χ^2^ = 1.96, *p*-value = 0.16), suggesting that the microchip tagging was not the cause of the unbalanced sex ratio we observed in our experiment.”

Considering those three points, we think it is not fair to say that we revised nothing in the manuscript, or maybe the referee got a wrong version of the manuscript.

The major criticism of the referee for the present version is the following:

**The most important conclusion is “early growth may be a cause rather than a consequence of sex determination in sea bass”. The authors stated that “The study was not focused exactly on sex differentiation progress”, but the conclusion is closely related to sex differentiation, or so-called “sex determination”. I do not see any results that will support this conclusion. Sex differentiation is a process. For European sea bass, it is a relatively long process. As illustrated in Vandeputte et al. (Eco & Evo, 2020), sex differentiation initiated as early as 33 DPH, which is earlier than sexual size dimorphism in the present work. Why authors draw opposite conclusions for the same group of authors?
Besides, the authors used the term “sex determination” to infer when sex is determined. However, the term “sex determination” is recognized as “what determines sex”, such as genetics or environments.**

First, as already stated before, “This leads to the hypothesis that early growth may (indeed now “might”) be a cause rather than a consequence of sex determination” is not a conclusion but an hypothesis which is compatible with the data in the paper and which we find interesting to highlight for future research. Please note that we also propose an alternative hypothesis, that differentiation between males and females starts earlier than what has been evidenced until now (line 322).

We feel that one of the key points of misunderstanding with the referee lies in the use of the words “sex determination” and “sex differentiation”. Our theoretical frame is that used, for example, by Devlin and Nagahama (Aquaculture, 208: 191-364, 2002) who mention “assignment of gonad determination as well as subsequent differentiation”. In this framework, which is generally accepted, vertebrates start their life with an undifferentiated gonad, which differentiates as male or female. If sex determination is purely genetic, sex is determined at conception, and there is no question of timing. If sex determination is environmental (or both environmental and genetic, as in sea bass), then timing is critical, and sex determination only happens once the individuals have received and integrated environmental (and eventually genetic) cues that direct them either towards male or towards female sex differentiation. This timing of sex determination, stricly speaking, is when differentiation is engaged towards one sex and cannot be reversed to the other sex. In the sea bass, it is widely recognized that sex is fully determined at ~80 mm body length, where morphological differentiation of the gonads becomes visible and sex cannot be changed anymore by temperature or administration of exogenous stroids (Piferrer et al., 2005, doi:10.1016/j.ygcen.2005.02.011). What we see in Vandeputte et al. (Ecol. Evol., 2020), is that indeed sex is not determined before that, as sex-ratio can be modified by temperature treatments until 230-244 days at 16°C, an age at which fish reach 75-80 mm.

Interestingly, molecular differentiation can be seen before this stage, although sex remains labile (thus not determined), as highlighted just before. Blazquez et al (2009 DOI: 10.1002/jez.b.21286) found that at ~50 mm body length, differential expression of *cyp19a1a* could be seen between future males and future females.

In the paper by Vandeputte et al. (Ecol. Evol., 2020), we did not demonstrate that “sex differentiation initiated as early as 33 dph”, as stated by the referee. We showed that temperature at 32 dph did have an impact on sex determination, and thus on sex differentiation at a much later time. More specifically, all treatments (control, switch at 32, 53, 74 dph) had similar expression levels of *cyp19a1a* and *gsdf* at 75 dph, and differences appeared only at 96 dph. This shows that expression differences can occur at 96 dph (but not 75 dph). However; although molecular differentiation seems to have started at 96 dph, sex is not yet determined, as is shown by the fact that the same control fish (reared at 16°C) which exhibit a higher expresssion of *gsdf* at 96 dph can end up 29% female when switched to 21°C at 104 dph or only 8.6% female when switched to 21°C at 230 dph. Thus, although molecular differentiation may appear around 96 dph, sex is not determined before a body length of 75-80 mm is reached. Our interpretation is thus that this molecular differentiation, which happens during the labile period, is not necessarily indicative of a definitive sexual fate of the gonad, but more of the integration of environmental and genetic cues affecting sex determinantion. Growth could play a role here especially through what has been proposed as the size threshold model for sex determination, proposed by Ospina -Alvarez and Piferrer (2008, <http://dx.doi.org/10.1371/journal.pone.0002837>), under which “when a critical time is reached during development, a sexually undifferentiated gonad will develop as an ovary or as a testis depending on whether it has attained a certain size above or below a threshold”.

We detailed this at lines 328-336, and again, please read this as a hypothesis, not as a conclusion. This is further highlighted at lines 336-339.
